# Fluorescent Polymer Incorporating Triazolyl Coumarin Units for Cu^2+^ Detection via Planarization of Ict-Based Fluorophore

**DOI:** 10.3390/s17091980

**Published:** 2017-08-30

**Authors:** Jean Marie Vianney Ngororabanga, Jacolien Du Plessis, Neliswa Mama

**Affiliations:** Department of Chemistry, Nelson Mandela Metropolitan University, Port Elizabeth 6031, South Africa; s212438700@live.nmmu.ac.za (J.M.V.N.); s215011619@live.nmmu.ac.za (J.D.P.)

**Keywords:** pendant group, triazolyl coumarin, chemosensor, aggregate, planarization

## Abstract

A novel fluorescent polymer with pendant triazolyl coumarin units was synthesized through radical polymerization. The polymer showed reasonable sensitivity and selectivity towards Cu^2+^ in acetonitrile in comparison to other tested metal ions with a significant quenching effect on fluorescence and blue shifting in the range of 20 nm. The blue shift was assigned to the conformation changes of the diethylamino group from the coumarin moiety which led to planarization of the triazolyl coumarin units. The possible binding modes for Cu^2+^ towards the polymer were determined through the comparison of the emission responses of the polymer, starting vinyl monomer and reference compound, and the triazole ring was identified as one of the possible binding sites for Cu^2+^. The detection limits of the polymer and vinyl monomer towards Cu^2+^ were determined from fluorescence titration experiments and a higher sensitivity (35 times) was observed for the polymer compared with its starting monomer.

## 1. Introduction 

The cupric ion (Cu^2+^) is considered as one of the trace elements in human and other mammal systems due to its essentiality and very limited quantities in the body [1,2]. Being ranked third after Fe^2+^ and Zn^2+^ in the human body as essential transition metal ions, Cu^2+^ concentrations beyond the necessary for biological functions can lead to oxidative stress and cellular toxicity, which are often associated with serious neurodegenerative diseases such as Alzheimer’s, Parkinson’s, and Wilson’s diseases [3,4,5]. In addition, the accumulation of copper in the environment due to the increased release of this metal from industrial activities presents ecological and human health threats [6,7]. For instance, an over-ingestion of copper can lead to serious health problems such as gastrointestinal disturbances and liver or kidney damage [7]. For these reasons, a limited concentration of 1.3 ppm (~20 µM) was set by the US Environmental Protection Agency (EPA) for copper in drinking water. Due to the widespread use of copper and the toxicity associated with its higher concentration, there is a strong need for reliable, inexpensive and simple methods for detecting and quantifying copper ions in different media for real-time monitoring of the environment, as well as biological and industrial samples. Typical methods that have been developed and employed for the detection of copper ions are mainly based on atomic absorption spectroscopy (AAS) [8], inductively coupled plasma-mass spectroscopy (ICP-MS), inductively coupled plasma-atomic emission spectroscopy (ICP-AES) [9], and electrochemical sensing methods [10,11]. However, these techniques are costly, extremely tedious and destructive. Furthermore, sample preparation requires large amounts and the methods are not suitable for continuous monitoring.

Due to their simplicity and high sensitivity, fluorescent-based methods showed some potential to address some problems faced by these methods, and several fluorescent chemosensors with different fluorophores have been developed. Owing to the excellent properties of the coumarin motif, such as biocompatibility, high fluorescence quantum yield, and relative ease of synthesis, absorption and emission tunability via substituent manipulation [12], the coumarin fluorophore is one of the widely used fluorophores in the synthesis of chemosensors for Cu^2+^. Nevertheless, most of the currently developed Cu^2+^ chemosensors are of low molecular weight and only a few qualify to be in the macromolecular range.

Owing to low chemical and thermal resistance as well as difficulties in separation and recovery associated with the low-molecular-weight chemosensors, a physical immobilization support is often needed for their application [13]. A physical support does not only improve the mechanical properties, but also minimizes the tendency of the sensing molecules to migrate. To avoid complications associated with synthesizing probes and immobilizing them on a physical support, polymers with host binding sites as part of their backbone or as part of their pendant group were found to be better alternatives. These materials have good thermal and mechanical properties and they also offer an outstanding and permanent immobilization method which allows them to be processed into end-user materials such as coatings and films [14,15,16,17,18]. Furthermore, fluorescent polymer-based chemosensors offer crucial advantages such as higher sensing performance levels (sensitivity and selectivity) compared to their small-molecule counterparts. So far, several polymer-based chemosensors for various metal ions have been developed [19,20,21], but very few showed selective sensitivity towards Cu^2+^ [22,23].

Herein we describe the chemosensing capability of a novel fluorescent triazolyl coumarin-based polymer (**P1**) towards metal ions. The 1,2,3-triazole ring as a receptor was firstly incorporated in a polymerizable vinyl monomer through Cu(I)-catalyzed azide-alkyne 1,3-dipolar cycloaddition (CuAAC), archetypal click reaction [24,25]. The absorption and emission properties of **P1** were studied in the presence of different metal ions and selective recognition with a fluorescence quenching response was observed in the presence of Cu^2+^. In addition to this, the presence of Cu^2+^ induced a remarkable blue shift in the emission spectrum of **P1**. Emission studies of the starting vinyl monomer and a reference coumarin-based molecule in the presence of Cu^2+^ were used to investigate the binding mode of **P1** towards Cu^2+^.

## 2. Experimental

### 2.1. Materials

All chemicals and solvents were purchased from Sigma-Aldrich or Merck and were used as received without further purification. The 7-(diethylamino)-3-nitro-2H-chromen-2-one (1), 3-amino-7-(diethylamino)-2H-chromen-2-one (2), 3-azido-7-(diethylamino)-2H-chromen-2-one (3), [26] compound **5** [26] and 7-(diethylamino)-2H-chromen-2-one [27] were prepared according to the literature. A stock solution of the polymer was prepared by dissolving the polymer in 25 mL acetonitrile to afford a solution of 3 × 10^−2^ g/mL. The solution was further diluted to 7 × 10^−5^. Deionized water was used to prepare solutions of metal ions and a concentration of 0.05 mol/L was obtained. All metal ion solutions were prepared using nitrate salts except for Fe^2+^ where sulphate was used. The titration experiments were performed using 3 mL of diluted acetonitrile polymer solution in a 3 mL quartz cuvette. In these experiments, spectroscopic measurements were taken after addition of an aliquot of selected metal ion solution. 

### 2.2. Measurement

^1^H NMR and ^13^C NMR spectra were recorded on a Bruker Avance DPX 400 (400 MHz) using TMS as an internal standard. FT-IR spectra were recorded in the range 4000–500 cm^−1^ using Opus software (version 6.5.6) on a Bruker Platinum Tensor 27 ATR-IR spectrophotometer. Size exclusion chromatography experiments were performed in DMAc at 40 °C (flow rate of 0.5 mL/min) using PMMA as a standard for calibration. The elemental analysis for carbon, hydrogen and nitrogen was performed using a Vario EL (Elementar Analysensystem GmbH) instrument. UV-Vis absorption and emission spectra were recorded at room temperature on a Perkin Elmer Lambda 35 and Perkin Elmer LS 45 respectively.

### 2.3. Polymer Synthesis 

#### 2.3.1. Synthesis of Compound 5

A mixture of 7-azido-4-methyl-2H-chromen-2-one (**4**) (0.1 g, 0.5 mmol), 3-butyn-2-ol (0.06 g, 0.5 mmol), CuSO_4_·5H_2_O (0.02 g, 0.054 mmol), sodium ascorbate (0.03 g, 0.15 mmol) and N,N,N’,N’’,N’’-pentamethyldiethylenetriamine (PMDETA) (0.03 g, 0.15 mmol) in tertahydrofuran (THF) (20 mL) was stirred at room temperature for 72 h. THF was removed under reduced pressure and the crude product dissolved in chloroform. The mixture was washed with water and then concentrated to afford a yellow solid product in 80% yield. m.p. 170–175 °C. IR νmax (cm^−1^): 3152 (C=C-H), 1719.73 (C=O), 1618 (C=C). ^1^H NMR (CDCl_3_, 400 MHz): δ = 8.83 (s, 1H), 8–7.98 (m, 2H), 6.47 (s, 1H), 5.46 (d, *J* = 4.72 Hz, 1H), 4.82 (q, *J* = 5.92, Hz, 1H), 2.48 (s, 3H), 1.49 (d, *J* = 6.48 Hz, 1H). ^13^C NMR (CDCl_3_, 400 MHz): δ = 159.95, 154.60, 154.13, 153. 24, 139.18, 127.68, 120.39, 119.73, 115.79, 115.03, 107.66, 61.98, 24.05, 18.56. Anal. Calc. for C_17_H_20_N_4_O_3_: C: 62.18, H: 6.14, N: 17.06. Found: C: 62.17, H: 6.30, N: 17.59.

#### 2.3.2. Synthesis of Monomer 6

In a two-necked round-bottomed flask equipped with a Dean Stark apparatus, a mixture of 7-(diethylamino)-3-(4-(1-hydroxyethyl)-1H-1,2,3-triazol-1-yl)-2H-chromen-2-one (**5**) (0.51 g, 1.54 mmol) and 20% para-toluenesulfonic acid (0.053 g, 0.31 mmol) in toluene (100 mL) were refluxed at 130 °C for 24 h. The reaction mixture was cooled to room temperature and then neutralized with sodium hydroxide solution (30 mL, 3 M). The organic layer was collected, washed with water (3 × 30 mL) and dried over anhydrous Na_2_SO_4_. The solvent was removed under reduced pressure and the crude product purified by column chromatography over silica gel (Hexane: EtOAc, 70:30) to afford a yellow solid in 50% yield. m.p. 149–151 °C. ^1^H NMR (CDCl_3_, 400 MHz): δ = 8.33 (s, 1 H), 7.33 (d, *J* = 8.52 Hz, 1H), 6.61 (d, *J* = 8.48 Hz, 1H), 6.48 (s, 1H), 5.92 (d, *J* = 17.6 Hz 1H), 5.32 (d, *J* = 11 Hz 1H), 8.48 (s, 1H), 3.38 (q, *J* = 6.36 Hz, 4H), 1.36 (d, *J* = 6.44 Hz, 3H), 1.17 (t, *J* = 6.75 Hz, 6H). ^13^C NMR (CDCl_3_, 400 MHz): δ = 156.92, 155.79, 151.56, 146.19, 134.48, 129.98, 125.45, 121, 116.84, 116.39, 110.08, 107.10, 97.03, 45, 12.4. Anal. Calc. for C_17_H_20_N_4_O_3_: C: 65.79, H: 5.85, N: 18.05. Found: C: 65.76, H: 6.24, N: 18.35.

#### 2.3.3. Synthesis of **P1**

To a 100 mL Schlenk flask, a mixture of vinyl monomer 6 (0.50 g, 1.61 mmol) and azobisisobutyronitrile (AIBN) (2 × 10^−2^ mmol) in dimethylformamide (DMF) (4 mL) was degassed using a freeze-thaw method (five cycles) followed by flushing with argon. The mixture was heated for 48 h at 70 °C and then poured to ethanol (50 mL). The precipitated polymer, after dropwise addition of a minimum amount of water, was filtered to afford polymers **P1** in 45% yield.

## 3. Results and Discussion

### 3.1. Synthesis and Characterization **P1**

**Scheme 1:**
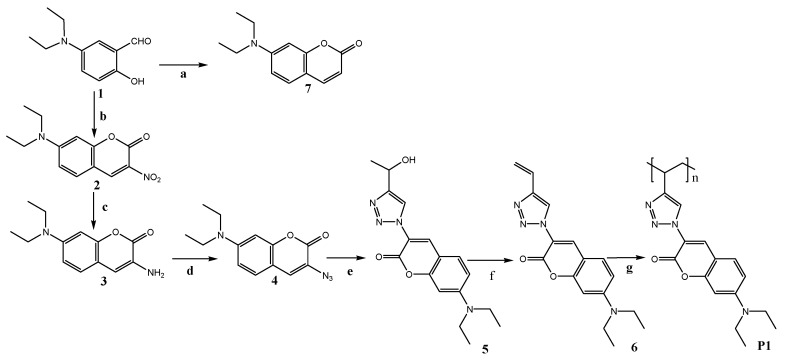
Synthesis of polymer **P1**; (**a**) Piperidine, ethanol, HCl, AcOH, reflux; (**b**) Ethyl 2-nitroacetate, AcOH, piperidine, Butanol, reflux; (**c**) SnCl_2_, HCl_aq_, rt; (**d**) NaNO_2_, KOAc, NaN_3_, HCl_aq_; (**e**) 3-Butyn-2-ol, CuSO_4_·5H_2_O, PMDETA, NaAsc, THF, rt; (**f**) PTSA, Toluene, 110 °C; (**g**) AIBN, DMF.

**Figure 1:**
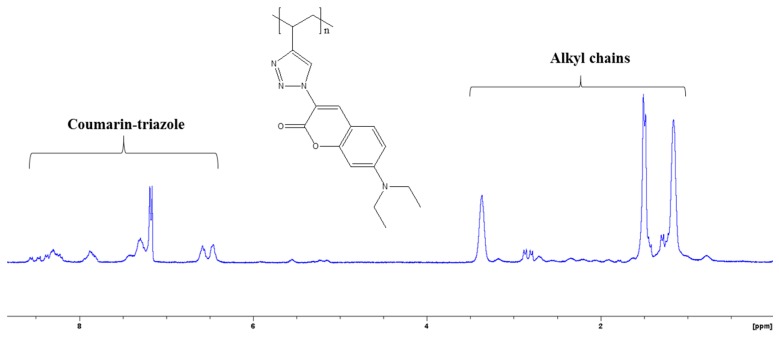
^1^H NMR spectrum for **P1** in CDCl_3_.

In order to incorporate a conjugated triazolyl coumarin unit into a polymer chain, a monomer with polymerizable functionality and a triazolyl coumarin unit which may act as both receptor and reporter was prepared. The synthesis was accomplished in five steps from 5-(diethylamino)-2-hydroxybenzaldehyde as shown in Scheme 1. The polymerization of monomer 6 was achieved through controlled radical polymerization, in which a degassed system was required to avoid oxygen interferences with the free radical species [28]. The structures of **P1** were confirmed by ^1^H NMR, as shown in Figure 1. Notable is the disappearance of the proton signals between 5.2 and 6.1 ppm in the monomer spectra (characteristic for the vinyl functionality), which are compensated by the appearance of the alkyl proton signals from the alkyl polymer backbone. The average molecular weight and polydispersity index of **P1** were determined to be 2.17 × 10^3^ and 1.92, respectively.

**P1** was partially soluble in less polar organic solvents and completely soluble in polar organic solvents such as acetonitrile, dimethyl sulfoxide (DMSO) and DMF. In order to avoid the interference of carbonyl and sulfinyl oxygen from DMF and DMSO solvents with the carbonyl group of the coumarin units, all spectroscopic measurements were performed in acetonitrile solvent. Detailed information regarding **P1** is summarized in Table 1 below.

### 3.2. Absorption Spectra Analysis

**Figure 2:**
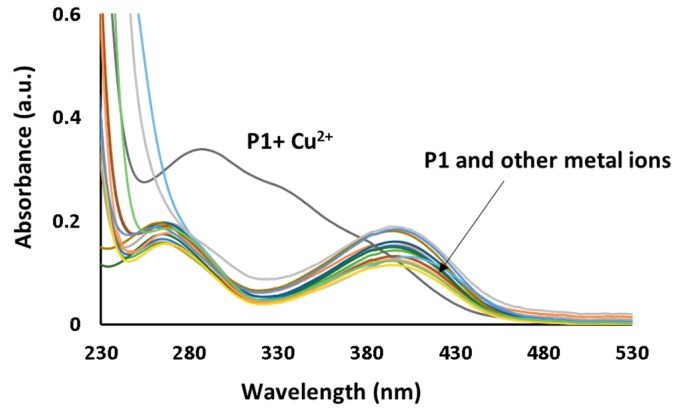
Absorption spectra of **P1** (4 × 10^−3^ g/L) in the presence of various metal ions (1.5 × 10^−4^ M aliquots) in acetonitrile.

The chemosensing capability of **P1** towards metal ions was initially investigated by UV-vis spectral analysis. This was achieved at room temperature in the presence of metal ions (monovalent, divalent and trivalent) such as Na^+^, Li^+^, Ca^2+^, Ag^+^, Al^3+^, Cr^3+^, Cu^2+^, Fe^2+^, Hg^2+^, Mn^2+^, Co^2+^, Zn^2+^, Cd^2+^, Ni^2+^ and Pb^2+^. As shown in Figure 2, all the metal ions except Cu^2+^ did not show any significant changes in the absorption peaks of **P1** at 265 nm and 396 nm. The addition of a Cu^2+^ aliquot induced an increase in the absorption peak intensity at 265 nm from 0.17 to 0.34 and a red shift to 287 nm. The absorption peak at 396 nm also showed a blue shift to 381 nm.

**Figure 3:**
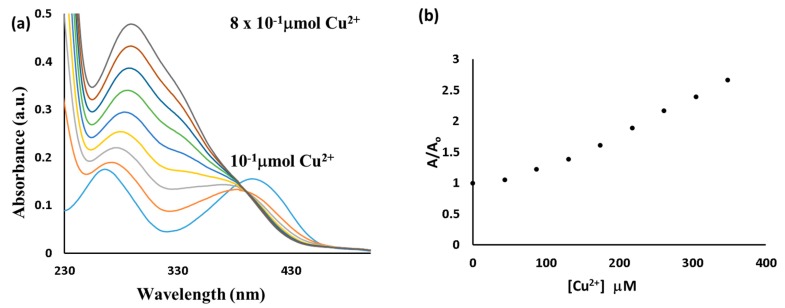
(**a**) Changes in the absorption spectrum of **P1** (4 × 10^−3^ g/L) in acetonitrile upon the addition of Cu^2+^ aliquots (0.05 M); (**b**) Plots of A/A_o_ against [Cu^2+^].

The titration of Cu^2+^ with **P1** (Figure 3a) showed a blue shift in the absorption peak at 396 nm and a gradual increase in the intensity, accompanied by a red shift in the absorption peak at 265 nm. A clear isosbestic point was observed at 390 nm, indicating a complex formation between **P1** and Cu^2+^. Using UV-vis titration data, a plot of A/A_o_ (where A_o_ and A are absorbance intensities in the absence and in the presence of Cu^2+^) against the concentration of Cu^2+^ was plotted (Figure 3b). Linearity between A/A_o_ and [Cu^2+^] was found in the 130–190 μmol/L range, with a correlation coefficient of R^2^ = 0.9989. The detection limit of **P1** was calculated according to the literature and was found to be 9 × 10^−6^ M [29].

### 3.3. Emission Spectra Analysis

**Figure 4:**
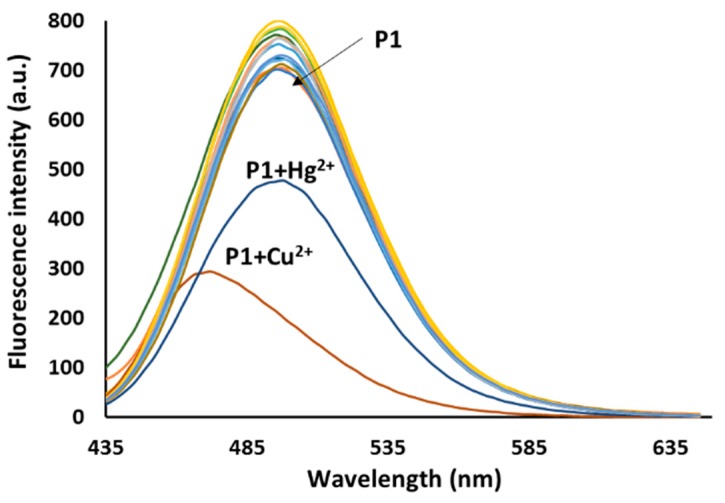
Emission spectra of **P1** (7 × 10^−5^ g/L) in the presence various metal ions (10 μL of 0.05 M solution) in acetonitrile. Excitation was performed at 395 nm.

The selectivity of **P1** towards various metal ions was also investigated using emission spectral analysis under identical conditions as the UV-vis spectral analysis. However, Hg^2+^and Cu^2+^ were the only metal ions that caused significant changes in the emission spectrum of **P1** when an aliquot of each tested metal ion was added to the acetonitrile solution of **P1** (Figure 4). The addition of Cu^2+^ caused a blue shift in the **P1** emission band from 484 to 469 nm, and a 68% intensity decrease, while the Hg^2+^ addition caused a 32% intensity decrease with no observable spectral shift. The fluorescent quenching in the presence of Hg^2+^and Cu^2+^ could be respectively attributed to the heavy metal effect [30,31] and the transfer of excitation energy from the fluorophores to the metal *d*-orbital or charge transfer from the fluorophores to the metal ion [32].

**Figure 5:**
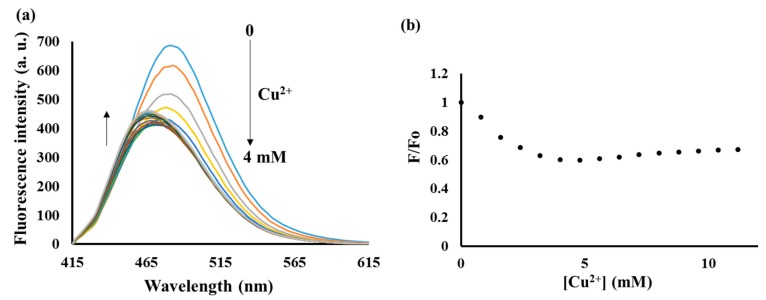
(**a**) Changes in the emission spectrum of **P1** (7 × 10^−5^ g/L) in acetonitrile upon the addition of Cu^2+^ aliquots (1.25 × 10^−3^ M); (**b**) plots of F/F_o_ against [Cu^2+^]. Excitation was performed at 395 nm.

The addition of Cu^2+^ aliquots to the **P1** solution from 0.8 to 4 μM led to a gradual decrease in the intensity of the emission peak at 484 nm which was accompanied by a blue shift from 484 to 470 nm (Figure 5a). Surprisingly, additional aliquots beyond 4 μM reversed the fluorescence response with a small increase in the intensity of the shifted emission band. The fluorescence increase was attributed to the aggregation-induced fluorescence mechanism (AIE) [33], from the aggregated triazolyl coumarin units resulting from the addition of Cu^2+^. This aggregation initiated the AIE process by restricting the intramolecular rotation of the diethylamino group which deactivates the excited states of fluorophores [34,35,36]. However, the process was not strongly expressed due to the presence of the highly quenching effect from Cu^2+^. The saturation point was achieved when the concentration of Cu^2+^ exceeded 10 μM. The maximum emission wavelength of the shifted peak at the saturation point was observed at 466 nm, suggesting a total shift of ca. 20 nm. A blue shift in the emission spectrum of **P1** suggests that the presence of Cu^2+^ induces the planarization in the triazolyl coumarin unit. In fact, dyes with electron donor, such as dialkylamino, and electron acceptor groups on the same aromatic ring form planar intermolecular charge transfer (ICT) structures with partial electron transfer upon electronic excitation. In polar environments, the dialkylamino group undergoes twisting which results in perpendicularity between the donor and acceptor orbitals [37]. This allows for the complete transfer of electrons and the mechanism is known as twisted intermolecular charge transfer (TICT) [38].

In **P1**, the addition of Cu^2+^ imposes greater spatial restriction in the triazolyl coumarin unit which leads to the planarization of the donor (diethylamino) and acceptor (carbonyl-triazole) groups in the excited state. This restricted planarization reduces the extent of conjugation which results in blue shifting of the **P1** emission band. Furthermore, it was reported that 7-dialkylaminocoumarins show a drastic reduction in the fluorescence quantum yields and fluorescence lifetimes in highly polar media due to the increased rate of the TICT process [34,35,36]. Since the addition of Cu^2+^ decreases the rate of TICT through planarization in triazoryl coumarin units, fluorescence quenching due to Cu^2+^ was opposed by the increasing quantum yield of triazoryl coumarin units which prevented the total quenching of the **P1** emission intensity upon the addition of Cu^2+^ aliquots. The detection limit was calculated from the plot of F/F_o_ (where F_o_ and F are fluorescence intensities in the absence and in the presence of Cu^2+^) against the concentration of Cu^2+^ (Figure 5b) in the linear range between 0–3.2 μM and was found to be 0.75 μM. This low detection limit compared to the detection limit obtained from absorption experiments highlights a higher sensitivity associated with fluorescence-based sensing systems [39,40], and is sufficiently low for the detection of Cu^2+^ at the sub-millimolar level.

**Figure 6:**
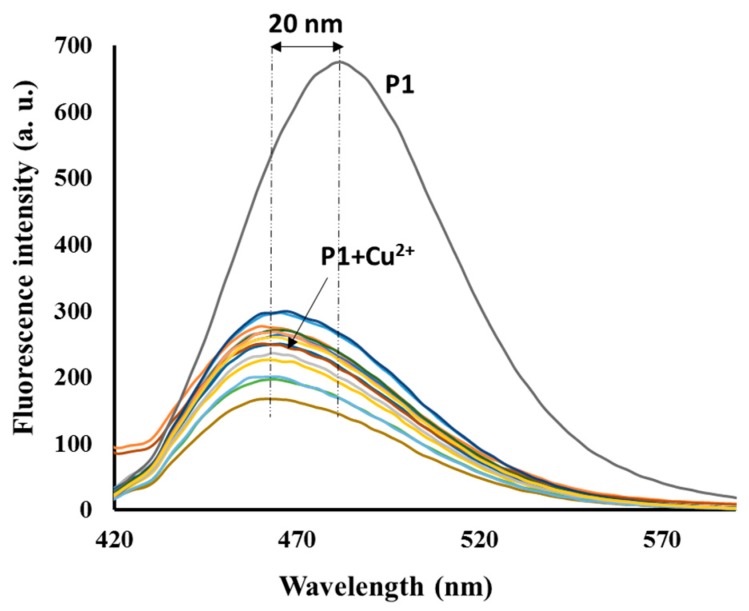
Fluorescence responses of **P1** (7 × 10^−5^ g/L) in the presence of a mixture of Cu^2+^ (1.5 × 10^−4^ M) and other metal ions (1.5 × 10^−4^ M). Excitation was performed at 395 nm.

To investigate the effect of other metal ions on the interaction between the **P1** and Cu^2+^, we conducted competitive studies with other metal ions in the presence of Cu^2+^. As shown in Figure 6, the fluorescence quenching and blue shift induced by the presence of Cu^2+^ ions in **P1** were not significantly affected by the presence of other metal ions. This indicates that **P1** and Cu^2+^ form a stable complex which cannot be interfered with the presence of other metal ions.

### 3.4. Sensing Mechanism of **P1** with Cu^2+^

**Figure 7:**
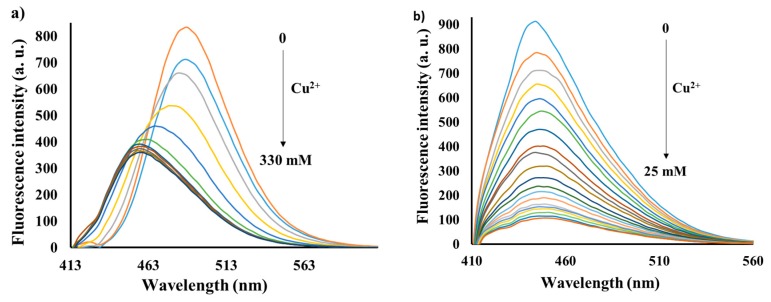
Changes in the emission spectra of (**a**) monomer 6 (5 × 10^−12^ M) and (**b**) compound 7 (3 × 10^−8^ M) in acetonitrile upon the addition of Cu^2+^ aliquots (monomer 6: 3 × 10^−5^ M Cu^2+^; compound 7: 1.3 μM Cu^2+^). Excitation was performed at 395 nm.

To study the binding mode between **P1** and Cu^2+^, the fluorescence responses of monomer 6 and compound 7 were investigated in the presence of increasing amounts of Cu^2+^ under the same conditions as **P1** spectral investigations. Similar to **P1**, the addition of Cu^2+^ aliquots to the monomer 6 solution induced a gradual decrease in intensity of the emission peak at 489 nm, accompanied by a blue shift from 489 to 458 nm (Figure 7a), while in compound 7 only a gradual decrease in emission intensity was observed (Figure 7b). These observations indicate that the triazole ring interacts with Cu^2+^ which leads to the planarization of the triazolyl coumarin unit in both monomer 6 and **P1**. Since compound 7 has only one possible binding site (the carbonyl group), the fluorescence quenching responses upon the addition of the increasing amount of Cu^2+^ indicate that the carbonyl group also takes part in Cu^2+^ binding. From the fluorescence titration experiments of monomer 6, a detection limit of 26 μM was calculated. This higher detection limit (~35 times higher than the **P1** detection limit) clearly indicates how the collective properties of the fluorophores in the polymer enhance the sensitivity in comparison to their monomer counterparts.

**Figure 8:**
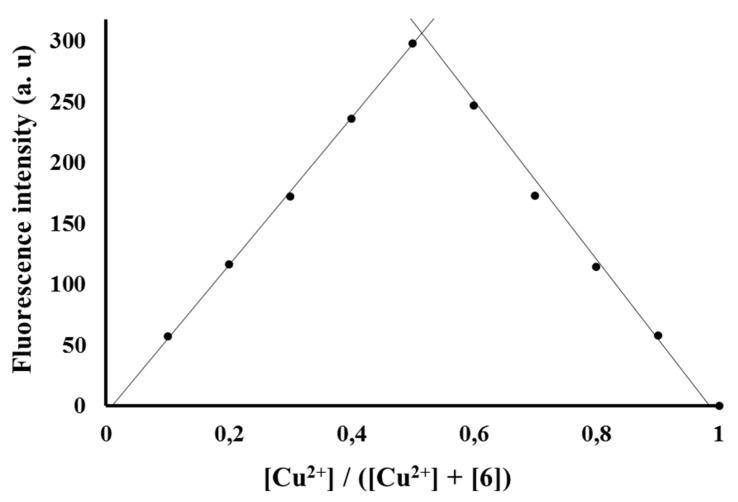
A job plot for monomer 6-Cu^2+^ complexation with a constant total concentration of 6.5 × 10^−11^ M in acetonitrile. Excitation was performed at 385 nm.

In order to determine the maximum number of triazole rings which take part in Cu^2+^ binding during complexation, a job plot analysis was carried out on monomer 6 using a continuous variation method [41]. The total concentration of added Cu^2+^ and monomer 6 was kept constant (6.5 × 10^−11^ M), and the plot of the emission intensity versus the molar fraction of Cu^2+^ at 395 nm is shown in Figure 8. Notably, the minimum emission intensity was achieved when the molar fraction was 0.5, which suggests a 1:1 stoichiometry of Cu^2+^:monomer 6 complexation. The association constant (K_a_) of **P1** with Cu^2+^ was evaluated graphically using the Benesi-Hildebrand equation (absorption method) [42,43,44]. The calculated value of K_a_ from the slope and intercept of the line was 2.6 × 10^3^ M.

**Scheme 2:**
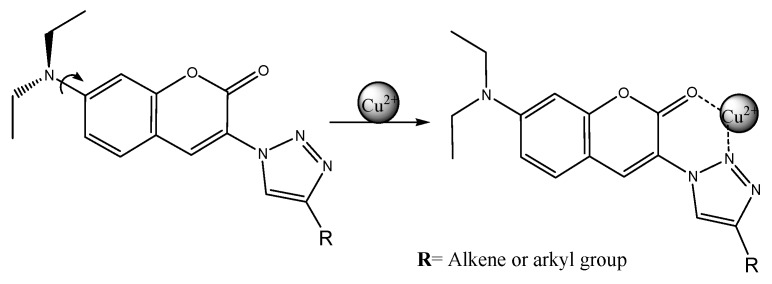
A schematic representation of the Cu^2+^-triazolyl coumarin unit interaction.

**Figure 9:**
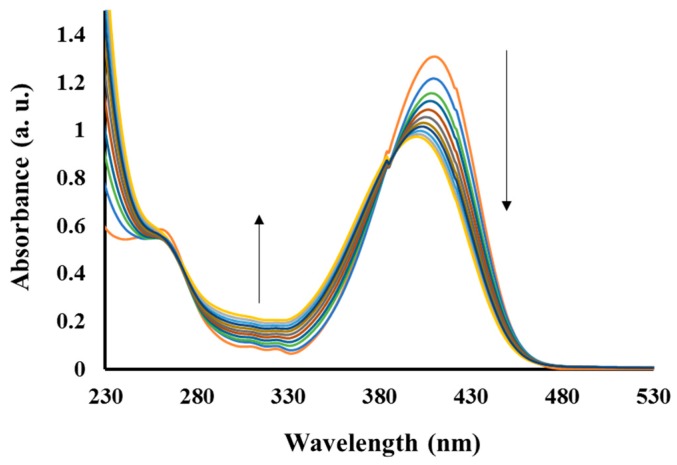
Changes in the absorption spectrum of monomer 6 (2 × 10^−5^ M) in acetonitrile upon the addition of Cu^2+^ aliquots (0.025 M).

Since the job plot analysis indicated a 1:1 stoichiometry of the Cu^2+^:monomer 6 complex, the interaction between Cu^2+^ and the triazolyl coumarin, which leads to blue shifting in the emission spectrum of **P1** and monomer 6, can be summarized in Scheme 2. To further analyze the Cu^2+^ interactions in monomer 6 and **P1**, UV-vis spectral analysis of monomer 6 in the presence of increasing amounts of Cu^2+^ was carried out (Figure 9). The addition of Cu^2+^ aliquots to the monomer 6 solution induced a gradual decrease in the intensity of the absorption peak at 412 nm, accompanied by a blue shift to 402 nm and a small absorption enhancement at ~300 nm. The blue shifting of the charge transfer absorption bands at 412 nm as in **P1** confirms the planarization of the triazoryl coumarin units. Furthermore, the comparison of the absorption spectra of **P1** and monomer 6 in the presence of increasing amounts of Cu^2+^ suggests that the planarization of triazoryl coumarin units in **P1** results in aggregation between the triazolyl coumarin units due to the increased π–π stacking. This was supported by a gradual increase in absorbance accompanied by the red shifting in the **P1** absorption band at 265 nm upon the addition of increasing amounts of Cu^2+^, which is characteristic of J-aggregate behavior [45]. This feature can be used to distinguish Cu^2+^ from other metal ions, as no other tested metal ions induced such a change in the absorption spectrum of **P1**.

### 3.5. Reversibility and Response Time

**Figure 10:**
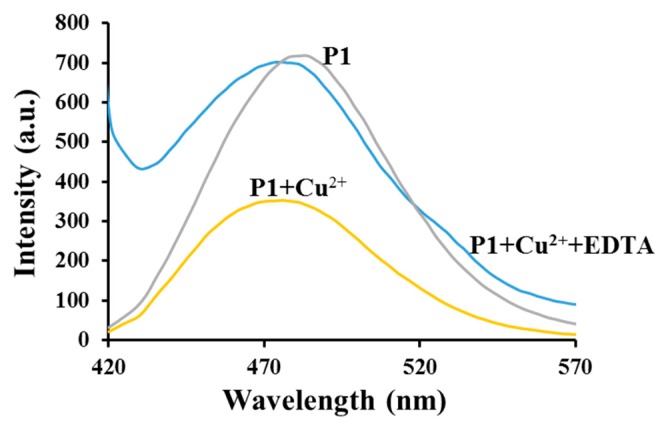
Reversibility of the interaction between **P1** and Cu^2+^ upon EDTA addition.

In order to examine the reversibility of **P1** complexation, the addition of EDTA to the acetonitrile solution of **P1** and Cu^2+^ was conducted (Figure 10). After the addition of an excess of EDTA to the solution, the fluorescence at 475 nm increased significantly and was again quenched upon the addition of Cu^2+^. These findings indicate that **P1** reversibly coordinates to Cu^2+^. Thus, it is plausible that **P1** may be recovered and reused in the laboratory and industrial settings. Although the fluorescence intensity of **P1** was regenerated upon the addition of EDTA, the blue spectral shift caused by the presence of Cu^2+^ could not be restored. This suggests that the complex that resulted from EDTA and Cu^2+^ also promotes the aggregation process of the triazolyl coumarin units. The course of the response of **P1** (7 × 10^−5^ to 1.7 × 10^−4^ g/L) in acetonitrile was also investigated. The results showed that the recognition interaction takes place in 30 s after the addition of Cu^2+^. Thus **P1** can be used for real-time monitoring of Cu^2+^ in practical analyses.

### 3.6. Preliminary Analytical Application

The applicability of the developed sensor was investigated in practical sample analysis using tap and lake water samples. The lake water samples were obtained from North End Lake (Eastern Cape, South Africa). In all samples analyzed using AAS, only tap water showed the presence of Cu^2+^ with the concentration of 0.275 ppm. The analytical studies of tap water samples using the developed sensor produced satisfactory results, as shown in Table 2. This indicates that **P1** could be used for the detection of Cu^2+^ even in complex media.

## 4. Conclusions

A novel polymer **P1** with triazolyl coumarin units as pendant groups was synthesized through multiple step syntheses. **P1** showed reasonable sensitivity and good selectivity towards Cu^2+^ in acetonitrile over a wide range of metal ions with remarkable quenching and blue shifting of the emission band. The blue shift in the emission band of **P1** was assigned to the planarization induced by the presence of Cu^2+^ in the triazolyl coumarin side chains and it was not interfered with by the presence of other metal ions. The investigation of the emission spectra of the starting monomer and reference compound 7 in the presence of Cu^2+^ indicated that the triazole ring and carbonyl functional groups were potential binding sites for Cu^2+^. From the comparison of the absorption behaviors of monomer 6 and **P1** in the presence of increasing amounts of Cu^2+^, it was noted that the planarization of triazolyl coumarin units in **P1** results in aggregation between the triazolyl coumarin units, which was supported by a red shift in the absorption spectrum of **P1**. The proposed sensor was also successfully applied in the determination of Cu^2+^ in real water samples. We believe that the design strategy, the remarkable photophysical properties of **P1** and the underlying mechanisms in the detection of Cu^2+^ will be useful in the development of novel polymer-based chemosensors which exploit new emerging signaling mechanisms.

## Figures and Tables

**Table 1:** Radical polymerization data and photophysical properties of polymer **P1**.

Polymer	M_w_ *	M_n_ *	PDI *	λ_abs_ (nm)	λ_ex_ (nm)	λ_emit_ (nm)	Stock Shift (nm)
**P1**	2.17 × 10^3^	1.19 × 10^3^	1.92	265 and 396	395	484	90

* Determined from SEC experiments (eluent: DMAc, PMMA standard).

**Table 2:** Determination of Cu^2+^ (ppm) in tap water.

Sample	1	2	3
Concentration (ppm)	0.255	0.317	0.328

## References

[1] Cowan J.A. (1997). Inorganic Biochemistry: An Introduction.

[2] Linder M.C., Azam M.H. (1996). Copper biochemistry and molecular biology. Am. J. Clin. Nutr..

[3] Multhaup G., Schlicksupp A., Hesse L., Beher D., Ruppert T., Masters C.L., Beyreuther K. (1996). The amyloid precursor protein of Alzheimer’s disease in the reduction of copper(II) to copper(I). Science.

[4] Deraeve C., Boldron C., Maraval A., Mazarguil H., Gornitzka H., Vendier L., Pitie M., Meunier B. (2008). Preparation and study of new poly-8-hydroxyquinoline chelators for an anti-Alzheimer strategy. Chem. Eur. J..

[5] Bruijn L.I., Miller T.M., Cleveland D.W. (2004). Unraveling the mechanisms involved in motor neuron degeneration in ALS. Annu. Rev. Neurosci..

[6] Waldermar M., Ryszard R., Teresa U. (1994). Effect of excess Cu on the photosynthetic apparatus of runner bean leaves treated at two different growth stages. Physiol. Plant..

[7] Georgopoulos P.G., Roy A., Yonone-Lioy M.J., Opiekun R.E., Lioy P.J. (2001). Environmental copper: Its dynamics and human exposure issues. J. Toxicol. Environ. Health Part B.

[8] Gonzales A.P.S., Firmino M.A., Nomura C.S., Rocha F.R.P., Oliveira P.V., Gaubeur I. (2009). Peat as a natural solid-phase for copper preconcentration and determination in a multicommuted flow system coupled to flame atomic absorption spectrometry. Anal. Chim. Acta.

[9] Liu Y., Liang P., Guo L. (2005). Nanometer titanium dioxide immobilized on silica gel as sorbent for preconcentration of metal ions prior to their determination by inductively coupled plasma atomic emission spectrometry. Talanta.

[10] Pathirathna P., Yang Y., Forzley K., McElmurry S.P., Hashemi P. (2012). Fast-scan deposition-stripping voltammetry at carbon-fiber microelectrodes: Real-time, subsecond, mercury free measurements of copper. Anal. Chem..

[11] Mülazımoğlu İ.E. (2012). Electrochemical determination of copper(II) ions at naringenin-modified glassy carbon electrode: Application in lake water sample. Desalin. Water Treat..

[12] Wheelock C.E. (1959). The Fluorescence of Some Coumarins1. Am. Chem. Soc..

[13] Yoshimura I., Miyahara Y., Kasagi N., Yamane H., Ojida A., Hamachi I. (2004). Molecular recognition in a supramolecular hydrogel to afford a semi-wet sensor chip. J. Am. Chem. Soc..

[14] Lv F., Feng X., Tang H., Liu L., Yang Q., Wang S. (2011). Development of film sensors based on conjugated polymers for copper(II) ion detection. Adv. Funct. Mat..

[15] García J.M., García F.C., Serna F., de la Peña J.L. (2010). High-performance aromatic polyamides. Prog. Polym. Sci..

[16] Anzenbacher P., Liuand Y., Kozelkova M.E. (2010). Hydrophilic polymer matrices in optical array sensing. Curr. Opin. Chem. Biol..

[17] Rotello V., Thayumanavan S., Rotello V., Thayumanavan S. (2008). Molecular Recognition and Polymers: Control of Polymer Structure and Self-Assembly.

[18] Gu C., Huang N., Wu Y., Xu H., Jiang D. (2015). Design of Highly Photofunctional Porous Polymer Films with Controlled Thickness and Prominent Microporosity. Angew. Chem. Int. Ed..

[19] Cui W., Wang L., Xiang G., Zhou L., An X., Cao D. (2015). A colorimetric and fluorescence “turn-off” chemosensor for the detection of silver ion based on a conjugated polymer containing 2, 3-di(pyridin-2-yl) quinoxaline. Sens. Actuators B Chem..

[20] Luo J., Jiang S., Qin S., Wu H., Wang Y., Jiang J., Liu X. (2011). Highly sensitive and selective turn-on fluorescent chemosensor for Hg^2+^ in pure water based on a rhodamine containing water-soluble copolymer. Sens. Actuators B Chem..

[21] Wang B., Liu X., Hu Y., Su Z. (2009). Synthesis and photophysical behavior of a water-soluble coumarin-bearing polymer for proton and Ni^2+^ ion sensing. Polym. Int..

[22] Guo Z.Q., Zhu W.H., Tian H. (2010). Hydrophilic copolymer bearing dicyanomethylene-4 H-pyran moiety as fluorescent film sensor for Cu^2+^ and pyrophosphate anion. Macromolecules.

[23] Dong Y., Koken B., Ma X., Wang L., Cheng Y., Zhu C. (2011). Polymer-based fluorescent sensor incorporating 2, 2′-bipyridyl and benzo[2,1,3]thiadiazole moieties for Cu^2+^ detection. Inorg. Chem. Commun..

[24] Rostovtsev V.V., Green L.G., Fokin V.V., Sharpless K.B. (2002). A stepwise huisgen cycloaddition process: Copper(I)-catalyzed regioselective “ligation” of azides and terminal alkynes. Angew. Chem. Int. Ed..

[25] Meldal M., Tornøe C.W. (2008). Cu-catalyzed azide-alkyne cycloaddition. Chem. Rev..

[26] Sivakumar K., Xie F., Cash B.M., Long S., Barnhill H.N., Wang Q. (2004). A fluorogenic 1, 3-dipolar cycloaddition reaction of 3-azidocoumarins and acetylenes. Org. Lett..

[27] Wu J., Liu W., Zhuang X., Wang F., Wang P., Tao S., Zhang X., Wu S., Lee S. (2007). Fluorescence turn on of coumarin derivatives by metal cations: A new signaling mechanism based on C=N isomerization. Org. Lett..

[28] Hasirci V., Yilgor P., Endogan T., Eke G., Hasirci N. (2011). Polymer fundamentals: Polymer synthesis. Compr. Biomater..

[29] Goswami S., Das A.K., Maity S. (2013). ‘PET’ vs. ‘push–pull’ induced ICT: A remarkable coumarinyl-appended pyrimidine based naked eye colorimetric and fluorimetric sensor for the detection of Hg^2+^ ions in aqueous media with test trip. Dalton Trans..

[30] McClure D.S. (1952). Spin-orbit interaction in aromatic molecules. J. Chem. Phys..

[31] Masuhara H., Shioyama H., Saito T., Hamada K., Yasoshima S., Mataga N. (1984). Fluorescence quenching mechanism of aromatic hydrocarbons by closed-shell heavy metal ions in aqueous and organic solutions. J. Phys. Chem..

[32] Wang R., Wan Q., Feng F., Bai Y. (2014). A novel coumarin-based fluorescence chemosensor for Fe^3+^. Chem. Res. Chin. Univ..

[33] Hong Y., Lam J.W., Tang B.Z. (2009). Aggregation-induced emission: Phenomenon, mechanism and applications. Chem. Commun..

[34] Satpati A.K., Kumbhakar M., Nath S., Pal H. (2009). Photophysical Properties of Coumarin-7 Dye: Role of twisted intramolecular charge transfer state in high polarity protic solvents. Photochem. Photobiol..

[35] Nag A., Bhattacharyya K. (1990). Role of twisted intramolecular charge transfer in the fluorescence sensitivity of biological probes: Diethylaminocoumarin laser dyes. Chem. Phys. Lett..

[36] Ramakrishna G., Ghosh H.N. (2002). Efficient electron injection from twisted intramolecular charge transfer (TICT) state of 7-diethyl amino coumarin 3-carboxylic acid (D-1421) dye to TiO2 nanoparticle. J. Phys. Chem. A.

[37] Sasaki S., Drummen G.P., Konishi G.I. (2016). Recent advances in twisted intramolecular charge transfer (TICT) fluorescence and related phenomena in materials chemistry. J. Mater. Chem. C.

[38] Nad S., Kumbhakar M., Pal H. (2003). Photophysical properties of coumarin-152 and coumarin-481 dyes: Unusual behavior in nonpolar and in higher polarity solvents. J. Phys. Chem. A.

[39] Kikuchi K.P.K. (2010). Design, synthesis and biological application of chemical probes for bio-imaging. Chem. Soc. Rev..

[40] Carter K.P., Young A.M., Palmer A.E. (2014). Fluorescent sensors for measuring metal ions in living systems. Chem. Rev..

[41] Renny J.S., Tomasevich L.L., Tallmadge E.H., Collum D.B. (2013). Method of continuous variations: Applications of job plots to the study of molecular associations in organometallic chemistry. Angew. Chem. Int. Ed..

[42] Benesi H.A., Hildebrand J.H. (1949). A spectrophotometric investigation of the interaction of iodine with aromatic hydrocarbons. J. Am. Chem. Soc..

[43] Barra M., Bohne C., Scaiano J.C. (1990). Effect of cyclodextrin complexation on the photochemistry of xanthone. Absolute measurement of the kinetics for triplet-state exit. J. Am. Chem. Soc..

[44] Wang L., Ye D., Cao D. (2012). A novel coumarin Schiff-base as a Ni(II) ion colorimetric sensor. Spectrochim. Acta Part A.

[45] Würthner F., Kaiser T.E., Saha-Möller C.R. (2011). J-Aggregates: From Serendipitous Discovery to Supramolecular Engineering of Functional Dye Materials. Angew. Chem. Int. Ed..

